# Low serum testosterone is associated with an increased risk of first-time renal calculi in men without testosterone replacement therapy

**DOI:** 10.1038/s41443-024-00963-x

**Published:** 2024-08-20

**Authors:** Austin Thompson, Danly Omil-Lima, Stephen Rhodes, Benjamin Jevnikar, Dana Obery, David Kaelber, Nannan Thirumavalavan

**Affiliations:** 1 Case Western Reserve University School of Medicine, Cleveland, OH USA; 2 Fox Chase Cancer Center at Temple University, Philadelphia, PA USA; 3 University Hospitals Cleveland Medical Center, Cleveland, OH USA; 4 Miami University, Oxford, OH USA; 5 The MetroHealth System, Cleveland, OH USA

**Keywords:** Urogenital diseases, Health care

## Abstract

The incidence of low serum testosterone has been increasing in men of all ages across a period which also corresponds to an increasing prevalence of kidney stones. Currently, the relationship between testosterone and kidney stones is unclear. Using the TriNetX Research Network, we performed a retrospective cohort study to evaluate the risk of developing an initial kidney stone in men based on their total testosterone level. Men aged ≥18 were divided into a low testosterone (<300 ng/dL) and normal testosterone (≥ 300 ng/dL) cohort. Men were excluded if they had a history of a kidney stone encounter diagnosis before testosterone measurement and a history of testosterone therapy prescription at any point. Propensity score matching was employed with an absolute standardized mean difference of less than 0.1 used as an indicator of successful matching. Our main outcome of interest was risk of developing an initial kidney stone in men aged ≥18 and within age-based subgroups. In men 18 and older, low testosterone was associated with a higher risk of one or more kidney stone encounter diagnoses (HR 1.12, 95% CI [1.09–1.15]). When stratified by age, no significant association between low testosterone and kidney stone encounter diagnoses was seen in men aged 18–24 (HR 1.09, 95% CI [0.85–1.39]). The highest risk was observed in men with low testosterone aged 34-44 (HR 1.29, 95% CI [1.17–1.38]). In this study, low serum testosterone was associated with an increased risk of initial kidney stone diagnosis in adult men without testosterone therapy prescriptions at any point in their life. Stratifying by age, the increased risk appears to begin in men aged 25, with the highest observed risk in men aged 33-44.

## Introduction

Testosterone is the hormone vital for the maintenance of secondary sex characteristics in men [1]. The maximum testosterone level a man experiences during their lifetime typically occurs between 20–25 years of age [2]. Serum testosterone levels are known to decline as men age [3, 4]. The proportion of men with low testosterone increases from 20% for men over 60, to 50% for men over 80 [3]. Recently, it has also been shown that serum testosterone levels have been declining on a population level since 1999 in adolescent and young adult males [5]. Symptoms of low testosterone levels include reduced sex drive, erectile dysfunction, sarcopenia, fatigue, depression, and difficulty with memory [6].

Known risk factors for kidney stones include obesity, diabetes, hypertension, metabolic syndrome, hot arid climate, urine electrolyte and pH imbalance [7]. Some of these risk factors are also associated with low testosterone. Medications including loop diuretics, acetazolamide, and topiramate have also been associated with kidney stone formation [8]. Recently, an association between testosterone replacement therapy and increased urinary stone disease was demonstrated [9].

The prevalence of kidney stones has increased worldwide since the 1990s [10]. Once an initial stone develops, kidney stone disease is highly recurrent with varying recurrence rates cited throughout the literature [11, 12]. Men are two times more likely than women to form kidney stones [13]. It is suspected that testosterone may contribute to the disparate rates of kidney stone formation between males and females. However, the role of testosterone in the development of kidney stones remains controversial.

In the present study, we utilize the multi-institutional TriNetX platform to investigate the association between testosterone levels and kidney stones in men aged 18 and older.

## Materials and methods

### Study design and database identification codes

We conducted a retrospective cohort analysis utilizing de-identified clinical information identified by querying the TriNetX Research Network (Cambridge, MA, USA). The STROBE (Strengthening the Reporting of Observational Studies in Epidemiology) guidelines were followed to the best of our ability within the confines of the TriNetX system when writing this manuscript [14]. We used the International Classification of Disease (ICD-10) and RxNorm codes to identify men for inclusion in this study (Supplemental Table 1). The main outcome of this study was one or more encounter diagnoses of kidney stones (calculus of the kidney), ICD-10 N20.0.

### Data source and patient selection

We used the TriNetX Research Network, a globally federated health research network to collect de-identified electronic health record (EHR) information compiled from 81 healthcare organizations (HCOs) across 4 countries. Because this study uses only de-identified EHR data, the MetroHealth System Institutional Review Board has deemed using TriNeX in this way exempt from IRB review. Clinical information for men included in this study was curated from 2007–2023. Statistical analyses were performed using the TriNetX platform on 1/7/2024. We selected males (aged ≥18) with a known testosterone level. Men with a kidney stone encounter diagnosis before testosterone measurement ((ICD-10 N20.0 (Calculus of kidney), Z87.442 (Personal history of urinary calculi)) and a history of a testosterone therapy (TTh) prescription ((RxNorm 10379 (testosterone), 37855 (testosterone 17-phenylpropionate)) at any point in their life were excluded. Body mass index (TriNetX Curated 9083, LOINC 39156-5) in this study represents a calculated value curated by the TriNetX research platform. Body mass index (BMI) categories representing underweight ( < 18.5 kg/m^2^), normal (18.5–24.9 kg/m^2^), overweight (25.0–29.9 kg/m^2^), obesity class I (30.0–34.9 kg/m^2^), obesity class II (35.0–39.9 kg/m^2^), and obesity class III ( > 40 kg/m^2^) were used at the time of propensity score matching. Age in this study represents the age at which men meet the index event, i.e., no kidney stone encounter diagnosis prior to their first laboratory-confirmed level of testosterone in addition to no history of a testosterone therapy prescription at any point in their life. Following American Urologic Association guidelines [15] we used a serum testosterone level of 300 ng/dL to divide the men into a low testosterone (< 300 ng/dL) and normal testosterone (300–1000 ng/dL) group. The collection time of testosterone levels is unknown in the TriNetX database. Demographic and clinical characteristics, including comorbidities and medications, were compared using descriptive statistics for all patients.

To delineate the association between low testosterone and kidney stones in men of different ages, we stratified men by age cohorts. Age strata included men aged 18–24, 25–33, 34–44, 45–54, 55–64, and ≥65. Men in these strata were matched to their eugonadal counterparts.

### Statistical analyses and data handling

To address potential confounders, we used propensity score matching based on patient demographic and clinical characteristics within 180 days prior to the index event (Table 1). We used 1:1 greedy nearest-neighbor matching with a caliper of 0.1 pooled standard deviations of the propensity score. An absolute standardized mean difference (SMD) of less than 0.1 for each variable included in the propensity score model indicated successful matching. Our outcome of interest was the risk of developing an initial kidney stone in men aged ≥18 following the first available laboratory-confirmed testosterone value. While testosterone deficiency guidelines [15] recommend using two testosterone values prior to initiating therapy, the purpose of our study was not related to therapy. This study excluded men with testosterone therapy receipt to investigate the association between low testosterone and kidney stones, and therefore a single testosterone value was used for inclusion in this study. Patients were followed until their last record in the TriNetX Research Network. Missing or partial data is not imputed or estimated by the TriNetX platform. Exceptions that may influence this study include discordance with HCO-supplied encounter dates and HCO encounter list. If an encounter is missing a start date, the earliest start data associated with the observations from the same encounter will be used. If an encounter is missing an end date, the latest end date from the observations associated with the encounter will be used. (https://trinetx.com/) Therefore, all patients included in this analysis should not have any missing data for our outcome of interest.

**Table 1:** Baseline patient characteristics for men ≥18 before and after propensity score matching.

Characteristic	Before Matching: Low Testosterone (*N* = 265,816)	Before Matching: Normal Testosterone (*N* = 530,406)	Before Matching: Standardized Mean Difference	After Matching: Low Testosterone (*N* = 263,557)	After Matching: Normal Testosterone (*N* = 263,557)	After Matching: Standardized Mean Difference
Age at Index (Mean ± SD)	53.4 ± 15.7	50.3 ± 15.2	0.20125	53.3 ± 15.7	52.9 ± 15.3	0.02315
Age at Index (18–24 years)	11,750	29,052	0.04875	11,739	11648	0.00168
Age at Index (25–33 years)	22,613	58,092	0.08258	22,594	22,582	0.00016
Age at Index (34–44 years)	42,828	100,534	0.07480	42,750	42,956	0.00212
Age at Index (45–54 years)	54,439	117,981	0.04304	54229	54,525	0.00278
Age at Index (55–64 years)	62,080	121,469	0.01075	61,648	61,882	0.00210
Age at Index (>65 years)	72,106	103,278	0.18182	70,597	69,964	0.00543
White	188,820	380,605	0.01600	187,289	187,014	0.00230
Not Hispanic or Latino	185,847	363,657	0.02933	183,950	183,751	0.00164
Black or African American	32,678	57,199	0.04726	32,181	32178	0.00003
BMI (Mean ± SD)	30.5 ± 6.2	28.6 ± 5.3	0.33901	30.3 ± 6.1	30.2 ± 5.9	0.02350
BMI ( < 18.5 kg/m2)	1365	2180	0.01511	1324	1297	0.00146
BMI (18.5–24.9 kg/m2)	12,273	33,456	0.07444	12,244	11,848	0.00719
BMI (25–29.9 kg/m2)	25,228	57,823	0.04664	25,115	24,947	0.00217
BMI (30–34.9 kg/m2)	20701	33,089	0.06070	20,259	20,716	0.00648
BMI (35–39.9 kg/m2)	10570	12,063	0.09794	9807	10,144	0.00670
BMI ( > 40 kg/m2)	5985	5158	0.10170	5209	5039	0.00467
Essential hypertension	59950	33,089	0.12514	58,238	59,019	0.00713
Diabetes mellitus	30880	93,100	0.14368	29,427	29,907	0.00576
Coronary artery disease	13527	18,357	0.08054	12,802	12,762	0.00071
Chronic kidney disease	10220	12,181	0.08983	9469	9511	0.00086
Heart failure	6361	6469	0.08819	5649	5475	0.00459
Gout	4797	6243	0.05181	4514	4548	0.00099
Cerebral infarction	2129	2730	0.03541	2002	1967	0.00154
Acute myocardial infarction	1811	2081	0.03955	1659	1543	0.00566
Pulmonary embolism	1674	1830	0.04089	1523	1451	0.00365
Acute deep vein thrombosis	1575	1720	0.03972	1424	1380	0.00230
Hyperparathyroidism and other disorders of parathyroid gland	668	1124	0.00819	646	648	0.00015
Crohn’s disease	643	1400	0.00439	637	617	0.00156
Bariatric surgery status	401	978	0.00820	398	382	0.00158
Family history of disorders of kidney and ureter	62	84	0.00535	57	52	0.00132
Hydrochlorothiazide	14707	21,789	0.06655	14,259	14,450	0.00319
Furosemide	9589	9124	0.11740	8505	8254	0.00543
Chlorthalidone	1542	2140	0.02525	1474	1466	0.00041
Topiramate	926	1582	0.00883	903	895	0.00052
Acetazolamide	387	489	0.01550	357	311	0.00491

*Each disease state represents an encounter diagnosis reported in the TriNetX Research Network

**Each medication represents a medication prescription reported in the TriNetX Research Network

Subgroup analyses were performed on age cohorts. Survival analysis was performed using a log-rank test and Cox proportional hazards regression. Unadjusted (before matching) and adjusted (after matching) hazard ratios (HR) and 95% confidence intervals are reported for each cohort. All analyses were performed on the TriNetX platform without any natural language processing.

## Results

### Patient characteristics before propensity score matching

The characteristics of all men aged ≥18 included in this study are seen in Table 1. 265,816 men with low testosterone and 530,406 men with normal testosterone were included. The mean ( ± standard deviation) age of men with low testosterone was 53.4 ± 15.7 years and 50.3 ± 15.2 years for men with normal testosterone (SMD 0.201). The age subgroup that differed significantly were men aged ≥65 (SMD 0.182). Comorbidities that differed significantly between the men with low testosterone compared to men with normal testosterone included BMI, encounter diagnoses for essential hypertension, and encounter diagnoses for diabetes mellitus. Furosemide use was the only medication prescription that differed significantly between the groups before propensity score matching.

When subgrouped by age, men aged 18–33 differed significantly in BMI (Supplemental Table 2, 3). Men aged 34–54 differed significantly in BMI, essential hypertension, and diabetes mellitus (Supplemental Table 4, 5). Men aged 34–54 differed significantly in BMI, essential hypertension, and diabetes mellitus, and furosemide use (Supplemental Table 6). Men aged ≥65 differed significantly in age at index, the number of African American men, BMI, diabetes mellitus, chronic kidney disease, heart failure, and furosemide use (Supplemental Table 7).

### Patient characteristics after propensity score matching

Propensity score matching established a cohort of 527,114 men aged ≥18 with a measured total serum testosterone level. 263,557 men with low testosterone were matched 1:1 to their eugonadal counterparts. The mean age of men with low testosterone was 53.3 ± 15.7 years whereas the mean age of men with normal testosterone was 52.9 ± 15.3 years. Once matched by propensity score matching, there were no large differences in covariates used for matching indicated by all absolute SMDs <0.1 (Table 1). Age strata for men with low testosterone included men aged 18–24 (*n* = 11,637), 25–33 (*n* = 23,582), 34-44 (*n* = 44,241), 45–54 (*n* = 56,748), 55–64 (*n* = 65,642), and ≥65 (*n* = 73,865). Men in these strata were matched 1:1 to their eugondal counterparts (Table 2).

**Table 2:** Number of patients with an incident kidney stone encounter diagnosis any time following a laboratory-confirmed serum testosterone level.

Age at Index	Low Testosterone (Total Number of Patients)**	Low Testosterone (Number of Patients Diagnosed with a Kidney Stone)	Low Testosterone (% of Cohort Diagnosed a Kidney Stone)	Normal Testosterone (Total Number of Patients)**	Normal Testosterone (Number of Patients Diagnosed with a Kidney Stone)	Normal Testosterone (% of Cohort Diagnosed with a Kidney Stone)	*p*-value*
≥18	263,557	10,817	4.10%	263,557	9,971	3.78%	<0.0001
18–24	11,637	131	1.13%	11,637	126	1.08%	0.515
25–33	23,582	448	1.90%	23,582	388	1.65%	0.040
34–44	44,241	1,248	2.82%	44,241	979	2.21%	<0.0001
45–54	56,748	2,314	4.08%	56,748	2,041	3.60%	<0.0001
55–64	65,642	3,410	5.19%	65,642	3,081	4.69%	<0.0001
≥65	73,865	4,082	5.53%	73,865	4,032	5.46%	0.004

*P-value from a log-rank test comparing survival curves across low and normal testosterone separately in each age cohort after propensity score matching.

**Sum of the subgroups is greater than the total of the ≥18 cohort because more patients are retained when matching within subgroups compared to the entire cohort ( ≥ 18 cohort).

### Kidney stone incidence increases with age regardless of serum testosterone levels

The number of men with low testosterone or normal testosterone with one or more kidney stone encounter diagnoses at any time following a laboratory-confirmed testosterone level in our matched cohorts is shown in Table 2. The proportion of men with one or more kidney stone encounter diagnosis increased with age in both the low testosterone and normal testosterone cohorts. In men aged 18 and older with low testosterone, 9684 (4.10%) men had a kidney stone encounter diagnosis compared to 8879 (3.78%) men with normal testosterone levels (*p* < 0.0001). Men 18–24 had the lowest proportion of kidney stones encounter diagnoses (1.13% low testosterone; 1.08% normal testosterone, *p* = 0.515), while men greater than 65 had the highest proportion of kidney stones encounter diagnoses (5.53% low testosterone; 5.46% normal testosterone, *p* < 0.004).

### Association of kidney stones with low testosterone in all men (aged 18 and older)

**Fig. 1:**
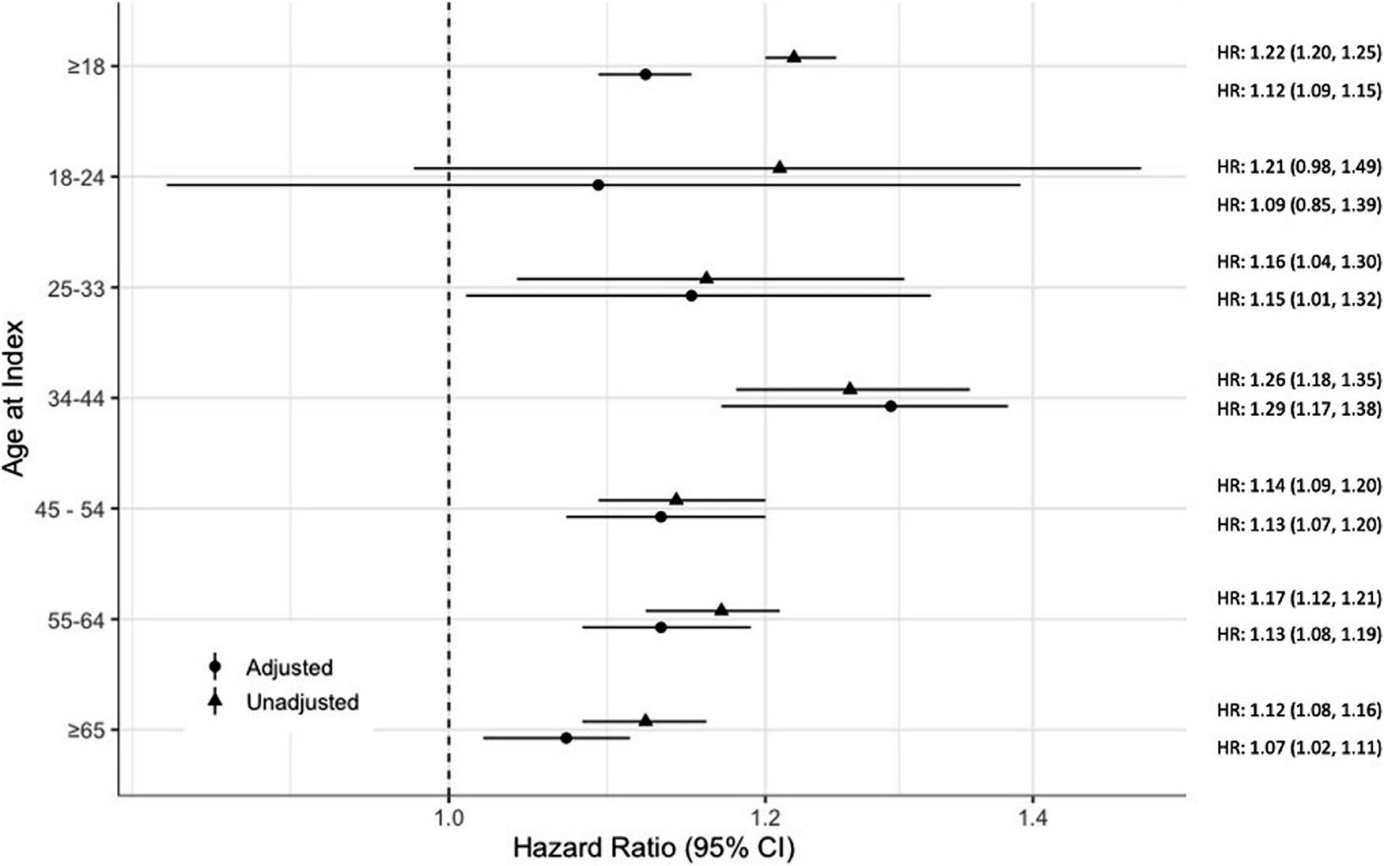
Hazard ratio for incident kidney stone encounter diagnosis any time following laboratory-confirmed low testosterone level compared to me with laboratory-confirmed normal testosterone. The total number of patients in each age subgroup is seen in Table 2.

For men 18 and older, low testosterone was associated with an increased risk of incident kidney stone encounter diagnosis compared to men with normal testosterone (adjusted HR: 1.12, 95% CI [1.09–1.15]) (Fig. 1).

### Association of kidney stones with low testosterone by age stratification

When stratified by age, men with low testosterone aged 25 and older had a statistically significant difference in the risk of initial kidney stone encounter diagnosis compared to men with normal testosterone (Table 2). Men 18–24 with low testosterone did not have a statistically significant difference in kidney stone encounter diagnoses (*p* = 0.515) (Table 2). Men aged 25-33 have an increased risk of encounter diagnoses of kidney stone compared to their eugonadal counterparts (adjusted HR: 1.15, 95% CI [1.01–1.32]) (Fig. 1). Men aged 34–44 with low testosterone had the highest risk of kidney stone encounter diagnosis when compared to their eugonadal counterparts (adjusted HR: 1.29, 95% CI [1.17–1.38]) (Fig. 1). Men aged 45–54 (adjusted HR: 1.13, 95% CI [1.07–1.20]), 55–64 (adjusted HR: 1.13, 95% CI [1.08–1.19]), and ≥65 (adjusted HR: 1.07, 95% CI [1.02–1.11]) have an increased risk of an encounter diagnosis of kidney stone compared to their eugonadal counterparts (Fig. 1).

## Discussion

In this study we used the American Urological Association’s testosterone cutoff of 300 ng/dL to investigate the association between low testosterone and incident diagnosis of kidney stones in 263,557 men with low testosterone [15]. To reduce the risk of confounding, men with low testosterone and normal testosterone were matched by age and BMI, along with comorbidities and medications associated with an increased risk of kidney stones. Our results show that an increased incidence of encounter diagnoses of kidney stones begins in men aged 25 with low testosterone and peaks in men aged 34–44 with low testosterone when compared to their eugonadal counterparts.

The prevalence of reported kidney stones in the National Health and Nutrition Examination Survey has increased dramatically since 1994 [16]. The prevalence of kidney stones increases with age, ranging from 5.1% to 19.7% for men aged 20–39 and greater than 80 years old, respectively [17]. Here, we showed that incident encounter diagnoses of kidney stones increased with age regardless of total testosterone status, but men with low testosterone were diagnosed more often than men with normal testosterone. To the best of our knowledge, no studies exist that investigate whether men with low testosterone experience more complications secondary to kidney stone presence or treatment compared to men with normal testosterone levels.

The excess risk of kidney stone development in men compared to women is not fully explained by differences in lifestyle risk factors [18]. Men are two times more likely than women to develop a kidney stone [13]. Predominant stone composition also differs between males and females. Men are more likely to develop calcium oxalate monohydrate stones whereas women are more likely to develop carbonate apatite and struvite stones [19]. Accordingly, men have been shown to excrete more calcium and oxalate per day than women [20]. To date, the role of testosterone in kidney stone formation is unclear.

Data supporting the role of testosterone in kidney stone development exists in animal models and human studies. Two studies posit that high testosterone levels are associated with kidney stones in men [21, 22]. However, these studies are limited by small sample sizes of 26 [21] and 40 [22] men. The physiologic basis underlying testosterone promotion of stone formation is unknown, but testosterone-induced hepatic glycolate oxidase activity and increased urinary excretion of oxalate have been shown in rats [23]. If transferrable to humans, a similar mechanism may underly the recent association of testosterone replacement therapy with an increased 2-year risk of stone events in hypogonadal men aged 40–64 [9].

Two studies have reported no significant association between testosterone levels and kidney stones [24, 25]. With a median follow-up length of 12.8 years, Knoedler et al. [24] reported no association between testosterone levels and all-time risk of kidney stones in men aged 40–79 living in Minnesota. A total of 102 men developed a kidney stone with 41 men developing an incident kidney stone following hormone measurement. No association between testosterone levels and incident kidney stones was seen in this study. Nackeeran et al. [25] also found no association between testosterone levels and a history of kidney stones in men aged 20 and older when analyzing the National Health and Nutrition Examination Survey (NHANES) [25]. Huang et al. [26] also showed no association between low testosterone and kidney stones in men aged 20–40 when analyzing NHANES [26].

In our study, we demonstrate that low testosterone is associated with an increased risk of incident kidney stone encounter diagnosis. Previously, two studies have shown an association between low testosterone levels and kidney stones [27, 28]. In men aged 41–60, testosterone levels were inversely associated with kidney stone incidence [26]. Importantly, we also showed an association between low testosterone and kidney stones in men aged 25–40. In studies showing an association between low testosterone and kidney stones, patients were either older than 35 [27] or no association was observed in men younger than 40 [26]. While more research is needed to confirm the association between low testosterone and kidney stones, it is important to consider increasing rates of hypogonadism in young adult males that may contribute to an increase in the prevalence of kidney stones in younger men.

Strengths of our study include a large sample size obtained from healthcare organizations from multiple geographic regions. We also employed an extensive list of comorbidities associated with kidney stone formation or low testosterone when matching. To the best of our knowledge, this is also the first study to directly include medications as an exclusion criterion or covariate for propensity score matching.

Limitations inherent to the use of curated EHR data containing de-identified data impact our study. First, we used ICD-10 and RxNorm codes to identify patients, which may contain errors. Second, in attempting to exclude patients with a history of kidney stones, it is possible that patients presented to healthcare organizations not included in the TriNetX platform and therefore have a history of kidney stones unknown to our data. This limitation is also transferrable to our attempt to exclude patients with testosterone therapy prescription at any point in their lives or identifying patients diagnosed with a kidney stone following inclusion in our study. Third, our study is likely not representative of men as a whole, given men were only included if they had a recorded testosterone level. Particularly, younger men may also be underrepresented in our study. Fourth, when splitting our cohort by testosterone values, we potentially discarded useful information inadvertently as a function of dichotomizing a continuous variable. Fourth, we could not control for urologist visits, which may introduce bias regarding the diagnosis frequency of kidney stones. Finally, our study strictly reports the association between low testosterone and renal calculi and thus limits the generalizability of these results to first-time renal stones. Future studies are needed to investigate the association between low testosterone and first-time urolithiasis, including ureteral stones.

The testosterone levels used in our study are also limited as TriNetX does not report the assay used or the time of collection. Given the increasing prevalence of hypogonadism and kidney stones, future studies using morning testosterone levels are needed as the association is currently unclear. Moreover, the dose-response relationship between total testosterone levels and incident kidney stones could also be investigated. Simultaneously, the association between testosterone therapy and kidney stones requires additional research as only one retrospective study has investigated this question. Future studies addressing these questions could inform future treatment of hypogonadal men to minimize the occurrence of kidney stones.

## Conclusion

In a cohort of 263,557 men aged 18 and older with low testosterone, we demonstrate that low testosterone ( < 300 ng/dL) is associated with an increased risk of first-time kidney stone encounter diagnoses in men without a history of kidney stones or testosterone therapy prescription when compared to men with normal testosterone. The increased risk appears to begin in men aged 25, with the highest risk of first-time kidney stones observed in men aged 34–44.

## Supplementary information

Supplemental Table 1. ICD-10 and RxNorm identification codes for covariants and outcome included in this study

Supplemental Table 2. Baseline patient characteristics for men aged 18-24 before and after propensity score matching

Supplemental Table 3. Baseline patient characteristics for men aged 25-33 before and after propensity score matching

Supplemental Table 4. Baseline patient characteristics for men aged 34-44 before and after propensity score matching

Supplemental Table 5. Baseline patient characteristics for men aged 45-54 before and after propensity score matching

Supplemental Table 6. Baseline patient characteristics for men aged 55-64 before and after propensity score matching

Supplemental Table 7. Baseline patient characteristics for men ≥65 before and after propensity score matching

## Data Availability

All data generated or analyzed during this study is included in this published article.
